# Biliary pharmacokinetic/pharmacodynamic analysis of continuous infusion meropenem/vaborbactam in a case series of orthotopic liver transplant recipients

**DOI:** 10.1093/jac/dkae261

**Published:** 2024-08-01

**Authors:** Milo Gatti, Matteo Rinaldi, Cristiana Laici, Simone Ambretti, Antonio Siniscalchi, Pierluigi Viale, Federico Pea

**Affiliations:** Department of Medical and Surgical Sciences, Alma Mater Studiorum University of Bologna, Bologna, Italy; Clinical Pharmacology Unit, Department for Integrated Infectious Risk Management, IRCCS Azienda Ospedaliero-Universitaria di Bologna, Via Massarenti 9, 40138, Bologna, Italy; Department of Medical and Surgical Sciences, Alma Mater Studiorum University of Bologna, Bologna, Italy; Infectious Diseases Unit, Department for Integrated Infectious Risk Management, IRCCS Azienda Ospedaliero-Universitaria di Bologna, Bologna, Italy; Division of Anesthesiology, Department of Anesthesia and Intensive Care, IRCCS Azienda Ospedaliero-Universitaria di Bologna, Bologna, Italy; Operative Unit of Microbiology, Department for Integrated Infectious Risk Management, IRCCS Azienda Ospedaliero-Universitaria di Bologna, Bologna, Italy; Division of Anesthesiology, Department of Anesthesia and Intensive Care, IRCCS Azienda Ospedaliero-Universitaria di Bologna, Bologna, Italy; Department of Medical and Surgical Sciences, Alma Mater Studiorum University of Bologna, Bologna, Italy; Infectious Diseases Unit, Department for Integrated Infectious Risk Management, IRCCS Azienda Ospedaliero-Universitaria di Bologna, Bologna, Italy; Department of Medical and Surgical Sciences, Alma Mater Studiorum University of Bologna, Bologna, Italy; Clinical Pharmacology Unit, Department for Integrated Infectious Risk Management, IRCCS Azienda Ospedaliero-Universitaria di Bologna, Via Massarenti 9, 40138, Bologna, Italy

## Abstract

**Objective:**

To analyse the biliary pharmacokinetics/pharmacodynamics (PK/PD) of continuous infusion (CI) meropenem-vaborbactam (MEM-VBM) in a case series of orthotopic liver transplant (OLT) recipients being treated for *Klebsiella pneumoniae* carbapenemase-producing *Klebsiella pneumoniae* (KPC-Kp) related biliary tract infections (BTIs) or as preemptive therapy of KPC-Kp rectal colonization.

**Methods:**

Critical OLT recipients receiving CI MEM-VBM (2 g/2 g q8h over 8 h) because of KPC-Kp related BTIs or as preemptive therapy of KPC-Kp rectal colonization, having Kehr’s tube positioned and undergoing simultaneous therapeutic drug monitoring of MEM and VBM in plasma and bile were retrospectively assessed. Bile-to-plasma ratio of free steady-state concentrations (*f*C_ss_) of MEM and VBM was used for assessing biliary penetration. Optimal joint MEM-VBM PK/PD target attainment was defined as MEM *f*C_ss_/MIC ratio >4 coupled with VBM free area under time–concentration curve (*f*AUC)/threshold concentration (C_T_) ratio >24.

**Results:**

Overall, four critical OLT recipients were included. Median bile-to-plasma ratio was 0.32 for MEM (range 0.21–0.79) and 0.40 for VBM (range 0.20–0.77). Biliary MEM-VBM joint PK/PD target attainment was optimal in 3/4 OLT recipients and quasi-optimal in the other one.

**Conclusions:**

The 1:1 proportion between MEM and VBM concentrations was maintained unchanged in the bile, allowing us to assume that the efficacy of MEM-VBM may be appropriate even in the treatment of BTIs. CI administration was an effective strategy for attaining aggressive biliary joint PK/PD targets against pathogens with an MIC up to 2 mg/L.

## Introduction

Gram-negative biliary tract infections (BTIs) are a common complication among orthotopic liver transplant (OLT) recipients,^1,2^ and the worrisome prevalence increase of carbapenemase-producing *Enterobacterales* (CPE) may affect both graft outcome and mortality risk.^3^ Additionally, rectal colonization by CPE may also increase the infection-related mortality risk among OLT recipients, requiring adequate preemptive therapy.^4^

Meropenem-vaborbactam (MEM-VBM) is a recently approved novel beta-lactam/beta-lactamase inhibitor combination (BL/BLIc) indicated for the treatment of intra-abdominal infections caused by *Klebsiella pneumoniae* carbapenemase-producing *Klebsiella pneumoniae* (KPC-Kp), including BTIs.^5^ Experimental models showed that the licensed dose of 2 g/2 g q8h over 3 h allowed to attain the minimum pharmacokinetic/pharmacodynamic (PK/PD) target of MEM/VBM required for granting efficacy against KPC-Kp with an MIC up to the EUCAST clinical breakpoint of 8 mg/L, namely 45%*f*T _> MIC_ of MEM in presence of a *f*AUC/MIC ratio of VBM > 24.^6^ However, it has been recently suggested that an aggressive MEM-VBM joint PK/PD target [i.e. a *f*C_ss_/MIC ratio of MEM ≥4 coupled with a *f*AUC/target concentration (C_T_) ratio of VBM >24] should be attained for maximizing clinical/microbiological efficacy and minimizing the risk of resistance development.^7,8^ In this scenario, continuous infusion (CI) administration could be helpful for this purpose.^7,8^

In deep-seated infections, aggressive PK/PD target should also be attained at the infection site.^9^ Good penetration rate in the biliary tract is a fundamental pharmacokinetic feature to be considered in the treatment of BTIs.^9^ Whereas MEM was recently shown to achieve therapeutically effective concentrations in the biliary tract of patients,^9^ information regarding VBM is currently lacking. Consequently, the right proportion of both components at the infection site has not been assessed yet.^10^

The aim of this study was to analyse the biliary PK/PD of CI MEM-VBM in a case series of OLT recipients affected by KPC-Kp BTIs or having KPC-Kp rectal colonization.

## Methods

Critically ill OLT recipients admitted in the period 1 September 2023 to 31 December 2023 at the post-transplant-ICU of the IRCCS Azienda Ospedaliero-Universitaria of Bologna, Italy, who received CI MEM-VBM because of KPC-Kp related BTIs or as preemptive therapy of KPC-Kp rectal colonization, had a Kehr’s tube positioned and underwent simultaneous therapeutic drug monitoring (TDM) of MEM and VBM in plasma and bile, were retrospectively included. KPC-Kp-related biliary infection was defined as the isolation of KPC-Kp in at least one bile sample collected from the Kehr’s tube or during the surgical intervention.^11^ Concomitant detection of KPC-Kp from at least one blood culture identified secondary bloodstream infection (BSI).^11^ KPC-Kp rectal colonization was defined as the isolation of KPC-Kp from a rectal swab in the absence of clinical signs and symptoms of infections.^12^

Antimicrobial susceptibility of MEM-VBM against KPC-Kp clinical isolates was tested by means of the broth microdilution method. The tested MIC values for MEM ranged from 0.008 to 128 mg/L in the presence of a fixed target VBM concentration of 8 mg/L. MIC values were interpreted according to the EUCAST guidelines, with a susceptibility clinical breakpoint of 8 mg/L.^13^

MEM-VBM commenced with a loading dose of 2 g/2 g administered over 2 h immediately followed by a maintenance dose of 2 g/2 g q8h over 8 h (namely by CI), according to stability in aqueous solution over time.^14^ Blood and bile samples for TDM of MEM-VBM were collected simultaneously after at least 24 hours from starting the therapy and reassessed subsequently whenever feasible. Total MEM and VBM steady-state concentrations (*C*_ss_) were measured by means of a validated liquid chromatography-tandem mass spectrometry method.^15^ The free fractions (*f*) of MEM and VBM were calculated by multiplying the total *C*_ss_ by 0.98 and 0.67, respectively, based on plasma protein binding of 2% and 33%.^16^

Aggressive joint PK/PD target was defined as optimal when both the MEM *f*C_ss_/MIC ratio was >4 and the VBM *f*AUC/C_T_ ratio was >24, quasi-optimal if only one of the two thresholds was achieved and suboptimal if neither of the two thresholds was achieved.^8,17^ The AUC of VBM was calculated by means of the following formula: AUC (mg·h/L) = dose (mg/24 h)/CL (L/h) [where CL was calculated as infusion rate (mg/h)/*C*_ss_ (mg/L)]. The C_T_ of VBM was 8 mg/L and corresponded to the fixed target concentration used by the EUCAST for the *in vitro* standard susceptibility testing of MEM-VBM.^13^ The MEM-VBM MIC values of the KPC-Kp clinical isolates were used for assessing the joint PK/PD target attainment. Microbiological eradication was defined as the elimination of the KPC-Kp strain from the primary site of infection or from the follow-up rectal swabs performed after ≥7 days from starting treatment.^8^

## Results

Overall, four OLT recipients receiving CI MEM-VBM were included (Table 1). Patient #1 [38 years; 130 kg; creatinine clearance (CL_CR_) 32 mL/min/1.73 m^2^] had a KPC-Kp related BTI (MIC = 0.25 mg/L) 28 days post-transplant. At the first TDM assessment, the biliary *f*C_ss_ was 10.09 mg/L for MEM (bile/plasma ratio = 0.26) and 22.45 mg/L for VBM (bile/plasma ratio = 0.41). The biliary joint PK/PD target attainment was more than optimal (MEM *f*C_ss_/MIC ratio = 40.38 and VBM *f*AUC/C_T _= 67.34). The MEM-VBM dosing regimen was reduced to 1 g/1 g q8h by CI. At TDM reassessment on day 3, the biliary *f*C_ss_ was 8.23 mg/L for MEM (bile/plasma ratio = 0.47) and 25.59 mg/L for VBM (bile/plasma ratio = 0.58). The biliary joint PK/PD target attainment was confirmed to be optimal (MEM *f*C_ss_/MIC ratio = 32.93 and VBM *f*AUC/C_T_ ratio = 76.78). Treatment duration was 14 days, and microbiological eradication and clinical cure were obtained.

**Table 1.** Bile and plasma steady-state concentrations, biliary penetration rate and PK/PD target attainment of MEM-VBM administered by CI in four OLT recipients as prophylaxis or targeted therapy against KPC-producing *Klebsiella pneumoniae* isolates

ID case	Age/gender	MEM/VBM MIC for KPC-Kp (mg/L)	Rationale for MEM/VBM treatment	MEM/VBM initial MD	Renal function	Overall TDM assessments	Average plasma MEM *f*Css (mg/L)	Average plasma VBM *f*Css (mg/L)	Average bile MEM *f*Css (mg/L)	Average bile VBM *f*Css (mg/L)	Average MEM bile/plasma ratio	Average VBM bile/plasma ratio	Average bile MEM *f*Css/MIC	Average bile VBM *f*AUC/C_T_	Joint PK/PD target on bile
#1	38/F	0.25	BTI	2 g/2 g q8h CI^a^	CL_CR_ 32 mL/min	2	28.27 ± 15.18	49.21 ± 7.63	9.16 ± 1.32	24.02 ± 2.23	0.36 ± 0.15	0.50 ± 0.12	36.65 ± 5.27	72.06 ± 6.68	Optimal
#2	48/M	0.5	BTI plus BSI	2 g/2 g q8h CI^a^	CVVHDF	2	93.74 ± 82.25	55.31 ± 41.17	20.43 ± 15.18	13.30 ± 4.78	0.24 ± 0.05	0.29 ± 0.13	40.87 ± 30.35	39.90 ± 14.35	Optimal
#3	48/M	2	Preemptive therapy due to rectal colonization	2 g/2 g q8h CI^b^	CL_CR_ 31 mL/min	1	17.05	54.94	6.37	20.84	0.37	0.38	3.19	62.51	Quasi-optimal
#4	42/M	0.064	Preemptive therapy due to rectal colonization	2 g/2 g q8h CI^b^	CL_CR_ 30 mL/min	1	86.63	77.39	68.50	59.97	0.79	0.77	1070.34	179.90	Optimal

CI, continuous infusion; CVVHDF, continuous veno-venous haemodiafiltration; MD, maintenance dose; MEM, meropenem; MEM/VBM, meropenem-vaborbactam; VBM, vaborbactam.

^a^Dosing adjustments based on a previous guidance^18^ recommending 50% dosing reduction when MEM *C*_ss_/MIC ratio was >10. In patient #2, dosing reduction >50% due to the finding of very high MEM *C*_ss_ linked to the potential risk of neurotoxicity (>64.2 mg/L)^18^

^b^No dosing adjustment due to preemptive therapy lasting only 48 h.

Patient #2 (48 years; 85 kg; anuric and undergoing continuous veno-venous hemodiafiltration; total effluent flow rate = 22.5 mL/kg/h; blood flow rate = 120 mL/min) had a BTI with a secondary BSI due to KPC-Kp (MIC = 0.5 mg/L) 18 day post-transplant. At day 1 after starting MEM-VBM, biliary *f*C_ss_ was 31.16 mg/L for MEM (bile/plasma ratio = 0.21) and 16.68 mg/L for VBM (bile/plasma ratio = 0.20). The joint PK/PD target was more than optimal (MEM *f*C_ss_/MIC ratio = 62.33 and VBM *f*AUC/C_T _= 50.05), so the MEM-VBM dosing regimen was reduced to 250 mg/250 mg q8h by CI. At TDM reassessment on day 5, biliary *f*C_ss_ was 9.70 mg/L for MEM (bile/plasma ratio = 0.27) and 9.92 mg/L for VBM (bile/plasma ratio = 0.38). The biliary joint PK/PD target attainment was confirmed to be optimal (MEM *f*C_ss_/MIC ratio = 19.40 and VBM *f*AUC/C_T _= 29.75), but unfortunately microbiological failure occurred and the patient passed away because of acute graft rejection after 27 days of treatment.

Patient #3 (48 years; 80 kg; CL_CR_ 31 mL/min/1.73 m^2^) had rectal colonization by KPC-Kp (MIC = 2 mg/L) and received preemptive therapy with CI MEM-VBM for 48 hours. At TDM assessment on day 1, biliary *f*C_ss_ was 6.37 mg/L for MEM (bile/plasma ratio = 0.37) and 20.84 mg/L for VBM (bile/plasma ratio = 0.38). The attained joint PK/PD target was quasi-optimal (MEM *f*C_ss_/MIC ratio = 3.19 and VBM *f*AUC/C_T_ ratio = 62.51). Follow-up rectal swabs after hospital discharge were negative.

Patient #4 (42 years; 85 kg; CL_CR_18 mL/min/1.73 m^2^) had rectal colonization by KPC-Kp (MIC = 0.064 mg/L) and received preemptive therapy with CI MEM-VBM for 48 hours. At TDM assessment on day 1, biliary *f*C_ss_ was 68.50 mg/L for MEM (bile/plasma ratio = 0.79) and 59.97 mg/L for VBM (bile/plasma ratio = 0.77). The attained joint PK/PD target was more than optimal (MEM *f*C_ss_/MIC ratio = 1070.34 and VBM *f*AUC/C_T_ ratio = 179.90). Follow-up rectal swabs after hospital discharge were negative.

## Discussion

Our case series first assessed the biliary penetration rates and the PK/PD target attainment of CI MEM-VBM in critically ill OLT recipients.

Our findings about the biliary penetration of MEM (median 0.32; range 0.21–0.79) after MEM-VBM CI administration are consistent with those of previous studies assessing MEM administration alone.^19,20^ In a PK study conducted among eight surgical patients, the mean MEM AUC bile-to-plasma ratio after a single 500 mg dose over 30 minutes infusion was 0.38.^19^ Interestingly, in our case series, the median and range (0.40; 0.20–0.77) of the VBM bile-to-plasma ratio was almost superimposable on that of MEM. The finding of a maintained 1:1 proportion between MEM and VBM concentrations in the bile is very valuable, allowing us to affirm that the PK/PD behaviour of this BL/BLIc may be appropriate even in the treatment of BTIs.

Joint PK/PD target attainment of both BL and BLI should be considered mandatory for granting efficacy with BL/BLIc.^7^ Notably, CI administration of MEM-VBM was revealed very effective for this purpose, as aggressive PK/PD target attainment was optimal in three cases and quasi-optimal in the other one. Real-time TDM was helpful in allowing us to reduce the MEM-VBM dosing regimen in the two cases with BTIs caused by very susceptible KPC-Kp. Conversely, the findings suggest that more intensified CI MEM-VBM dosing regimens could be needed for attaining optimal joint PK/PD target in the treatment of BTIs caused by KPC-Kp having borderline susceptibility.

Limitations of this study should be acknowledged. The limited sample size did not allow for a large-scale applicability. It could not be ruled out that the protein binding rates of MEM and of VBM into the bile could differ from those in plasma.

In conclusion, our PK/PD findings may allow the assumption that adequate efficacy of MEM-VBT may be granted even in the treatment of KPC-Kp-related BTIs. CI administration may be very effective in attaining optimal aggressive MEM-VAB joint PK/PD targets in the bile against pathogens with an MIC up to <2 mg/L. Further evidence is warranted for confirming our preliminary findings.

## Transparency declarations

M.G. reports grants from Angelini S.p.A., outside the submitted work. P.V. has served as a consultant for bioMérieux, Gilead, Merck Sharp & Dohme, Nabriva, Nordic Pharma, Pfizer, Thermo-Fisher and Venatorx, and received payment for serving on the speaker’s bureaus for Correvio, Gilead, Merck Sharp & Dohme, Nordic Pharma and Pfizer, outside the submitted work. F.P. participated in speaker bureau for Angelini, BeiGene, Gilead, InfectoPharm, Menarini, Merck Sharp & Dohme, Pfizer and Shionogi, and on the advisory board for BeiGene, Merck Sharp & Dohme, Pfizer and Viatris, outside the submitted work. The other authors report no potential conflicts of interest for this work.

## References

[1] Van Delden C . Bacterial biliary tract infections in liver transplant recipients. Curr Opin Organ Transplant 2014; 19: 223–8. 10.1097/MOT.0000000000000083 24752064

[2] Antunes M, Teixeira A, Fortuna P et al Infections after liver transplantation: a retrospective, single-center study. Transplant Proc 2015; 47: 1019–24. 10.1016/j.transproceed.2015.03.009 26036509

[3] Giannella M, Rinaldi M, Viale P. Antimicrobial resistance in organ transplant recipients. Infect Dis Clin North Am 2023; 37: 515–37. 10.1016/j.idc.2023.04.001 37244806

[4] Giannella M, Freire M, Rinaldi M et al Development of a risk prediction model for carbapenem-resistant *Enterobacteriaceae* infection after liver transplantation: a multinational cohort study. Clin Infect Dis 2021; 73: e955–66. 10.1093/cid/ciab109 33564840

[5] Paul M, Carrara E, Retamar P et al European Society of Clinical Microbiology and Infectious Diseases (ESCMID) guidelines for the treatment of infections caused by multidrug-resistant Gram-negative bacilli (endorsed by European Society of Intensive Care Medicine). Clin Microbiol Infect 2022; 28: 521–47. 10.1016/j.cmi.2021.11.025 34923128

[6] Griffith DC, Sabet M, Tarazi Z et al Pharmacokinetics/pharmacodynamics of vaborbactam, a novel beta-lactamase inhibitor, in combination with meropenem. Antimicrob Agents Chemother 2019; 63: e01659-18. 10.1128/AAC.01659-18 30397063 PMC6325214

[7] Gatti M, Pea F. Jumping into the future: overcoming pharmacokinetic/pharmacodynamic hurdles to optimize the treatment of severe difficult to treat-Gram-negative infections with novel beta-lactams. Expert Rev Anti Infect Ther 2023; 21: 149–66. 10.1080/14787210.2023.2169131 36655779

[8] Gatti M, Rinaldi M, Gaibani P et al A descriptive pharmacokinetic/pharmacodynamic analysis of continuous infusion meropenem/vaborbactam in the treatment of a case series of critically ill patients with documented KPC-producing *Klebsiella pneumoniae* ventilator-associated pneumonia. Int J Antimicrob Agents 2023; 62: 106992. 10.1016/j.ijantimicag.2023.106992 37778429

[9] Thabit AK . Antibiotics in the biliary tract: a review of the pharmacokinetics and clinical outcomes of antibiotics penetrating the bile and gallbladder wall. Pharmacotherapy 2020; 40: 672–91. 10.1002/phar.2431 32485056

[10] Gatti M, Viale P, Pea F. Therapeutic drug monitoring of ceftazidime/avibactam: why one leg is not enough to run. J Antimicrob Chemother 2024; 79: 195–9. 10.1093/jac/dkad367 38019676

[11] Horan TC, Andrus M, Dudeck MA. CDC/NHSN surveillance definition of health care-associated infection and criteria for specific types of infections in the acute care setting. Am J Infect Control 2008; 36: 309–32. 10.1016/j.ajic.2008.03.002 18538699

[12] Cano Á, Gutiérrez-Gutiérrez B, Machuca I et al Association between timing of colonization and risk of developing *Klebsiella pneumoniae* carbapenemase-producing K*. pneumoniae* infection in hospitalized patients. Microbiol Spectr 2022; 10: e01970-21. 10.1128/spectrum.01970-21 PMC9045231 35323035

[13] EUCAST . 2024. Meropenem-vaborbactam: rationale for EUCAST clinical breakpoints. https://www.eucast.org/fileadmin/src/media/PDFs/EUCAST_files/Rationale_documents/Meropenem-vaborbactam_Rationale_Document_v_2.0_202309xx_AM2.pdf

[14] Chen IH, Martin EK, Nicolau DP et al Assessment of meropenem and vaborbactam room temperature and refrigerated stability in polyvinyl chloride bags and elastomeric devices. Clin Ther 2020; 42: 606–13. 10.1016/j.clinthera.2020.01.021 32139176

[15] Barone R, Conti M, Giorgi B et al Fast and sensitive method for simultaneous quantification of meropenem and vaborbactam in human plasma microsamples by liquid chromatography-tandem mass spectrometry for therapeutic drug monitoring. Antibiotics (Basel) 2023; 12: 719. 10.3390/antibiotics12040719 37107082 PMC10135283

[16] Griffith DC, Loutit JS, Morgan EE et al Phase 1 study of the safety, tolerability, and pharmacokinetics of the β-lactamase inhibitor vaborbactam (RPX7009) in healthy adult subjects. Antimicrob Agents Chemother 2016; 60: 6326–32. 10.1128/AAC.00568-16 27527080 PMC5038296

[17] Gatti M, Rinaldi M, Laici C et al Role of a real-time TDM-based expert clinical pharmacological advice program in optimizing the early pharmacokinetic/pharmacodynamic target attainment of continuous infusion beta-lactams among orthotopic liver transplant recipients with documented or suspected gram-negative infections. Antibiotics (Basel) 2023; 12: 1599. 10.3390/antibiotics12111599 37998801 PMC10668725

[18] Gatti M, Cojutti PG, Bartoletti M et al Expert clinical pharmacological advice may make an antimicrobial TDM program for emerging candidates more clinically useful in tailoring therapy of critically ill patients. Crit Care 2022; 26: 178. 10.1186/s13054-022-04050-9 35701812 PMC9199203

[19] Ikawa K, Nakashima A, Morikawa N et al Clinical pharmacokinetics of meropenem and biapenem in bile and dosing considerations for biliary tract infections based on site-specific pharmacodynamic target attainment. Antimicrob Agents Chemother 2011; 55: 5609–15. 10.1128/AAC.00497-11 21947393 PMC3232762

[20] Granai F, Smart HL, Triger DR. A study of the penetration of meropenem into bile using endoscopic retrograde cholangiography. J Antimicrob Chemother 1992; 29: 711–8. 10.1093/jac/29.6.711 1506351

